# A novel indel variant in *LDLR* responsible for familial hypercholesterolemia in a Chinese family

**DOI:** 10.1371/journal.pone.0189316

**Published:** 2017-12-11

**Authors:** Hongyan Shu, Jingwei Chi, Jing Li, Wei Zhang, Wenshan Lv, Jie Wang, Yujie Deng, Xu Hou, Yangang Wang

**Affiliations:** 1 Department of Endocrinology, The Affiliated Hospital of Qingdao University, Qingdao University, Qingdao, China; 2 Department of Endocrinology, Linzi District People’s Hospital, Zibo, China; 3 Department of Gerontology, Linzi District People’s Hospital, Zibo, China; 4 Department of Thoracic Surgery, Linzi District People’s Hospital, Zibo, China; Hokkaido Daigaku, JAPAN

## Abstract

Familial hypercholesterolemia (FH) is an inherited disorder characterized by elevation of serum cholesterol bound to low-density lipoprotein. Mutations in *LDLR* are the major factors responsible for FH. In this study, we recruited a four-generation Chinese family with FH and identified the clinical features of hypercholesterolemia. All affected individuals shared a novel indel mutation (c.1885_1889delinsGATCATCAACC) in exon 13 of *LDLR*. The mutation segregated with the hypercholesterolemia phenotype in the family. To analyze the function of the indel, we established stable clones of mutant and wild-type *LDLR* in Hep G2 cells. The mutant LDLR was retained in the endoplasmic reticulum (ER) and failed to glycosylate via the Golgi. Moreover, the membrane LDLR was reduced and lost the ability to take up LDL. Our data also expand the spectrum of known *LDLR* mutations.

## Introduction

Familial hypercholesterolemia (FH, MIM 143890) is an inherited disorder characterized by elevated serum low-density lipoprotein (LDL) cholesterol levels, which result in excess deposition of cholesterol in tissues, leading to accelerated atherosclerosis and increased risk of premature coronary heart disease [1]. FH is primarily an autosomal dominant disorder, commonly caused by the mutations in the low-density lipoprotein receptor (*LDLR*), its ligand apoB (*APOB*), or proprotein convertase subtilisin–kexin type 9 (*PCSK9*) genes [2–4]. Recently, the apolipoprotein E (*AOPE*) mutation was found to be associated with dominant FH [5, 6]. An autosomal recessive form of FH usually is caused by loss-of-function mutations in LDLRAP1, which encodes a protein required for clathrin-mediated internalization of the LDL receptor [7]. The prevalence of heterozygous FH is estimated to be ~1:200–300 and that of homozygous FH is about 1:160,000–300,000 [8]. Without appropriate preventive efforts, approximately 85% of males and 50% of females with FH will suffer a coronary event before they reach 65 years old [9].

Mutations in the *LDLR* gene have been reported across the world. They are the major causes of FH, which has an autosomal dominant pattern [10]. *LDLR*, located at chromosome 19p13.2, is composed of 18 exons spanning 45 kb. The transcript of *LDLR* is 5.3 kb long, which encodes a peptide of 860 residues [11]. The LDL receptor mediates the endocytosis of cholesterol-rich LDL and thus maintains the plasma level of LDL [12]. Based on the LDLR protein domain that the mutations are localized, there are five broad classes of mutation, among which, class 2 mutations prevent proper transport to the Golgi body needed for modifications to the receptor [1].

We recruited a four-generation Chinese family with FH. We identified a novel indel variant, specifically c.1885_1889delinsGATCATCAACC, in exon 13 of *LDLR* after direct sequencing. This change led to a newly formed mutant p.Phe629_ser630delinsAspHisGlnPro, which is a class 2 mutation. The mutant LDLR failed to transport to the Golgi body, and the LDLR in cytomembrane was reduced. Meanwhile, the mutant LDLR lost the ability of uptake LDL.

## Materials and methods

### Study subjects

**Fig 1:**
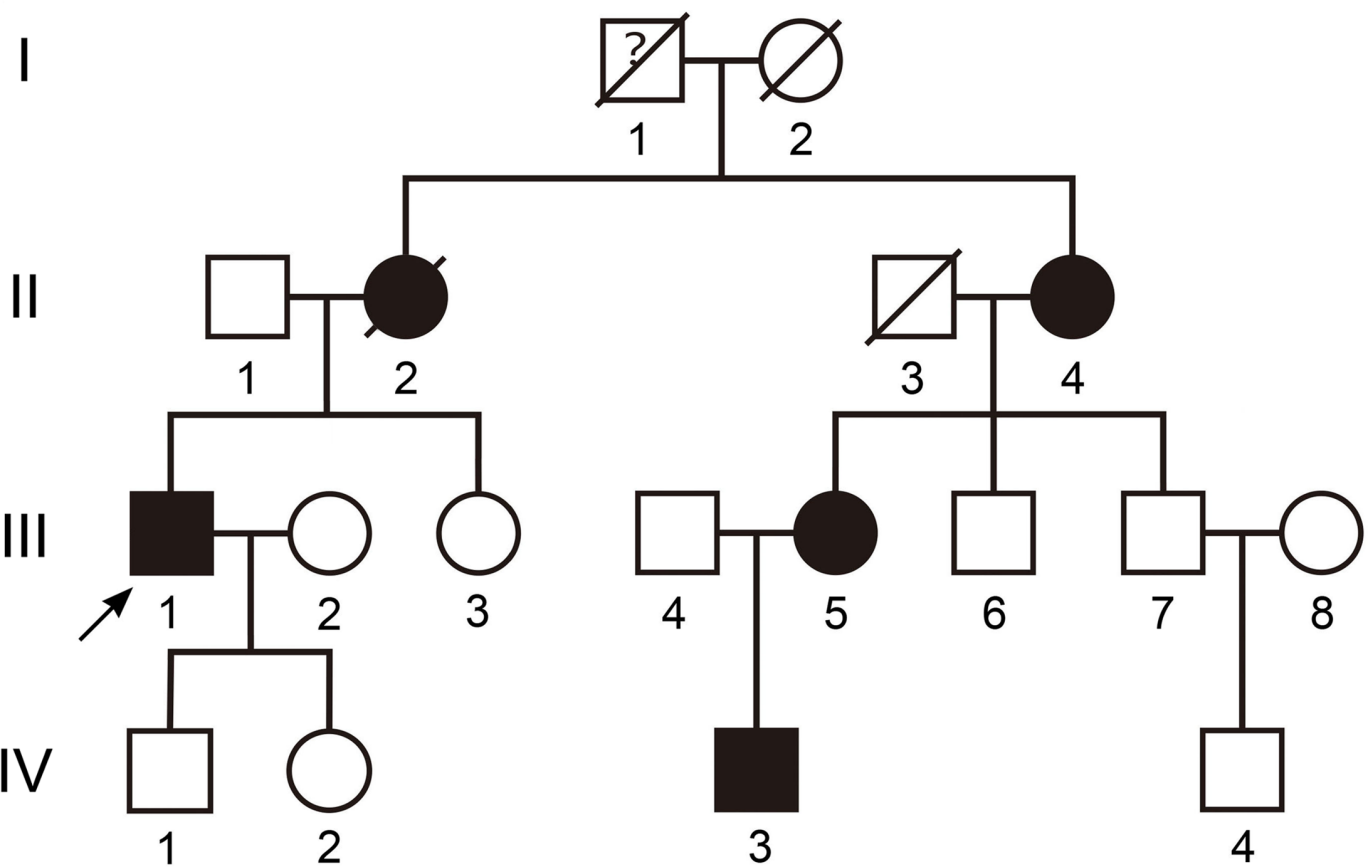
Pedigree of a Chinese family with familial hypercholesterolemia. Squares and circles indicate males and females, respectively. Black symbols represent individuals with a hypercholesterolemia phenotype and open symbols represent unaffected individuals. The proband is marked by an arrow.

We recruited a four-generation Han Chinese family with FH from the Affiliated Hospital of Qingdao University in 2015. In total, 14 family members participated in this study, including 4 affected individuals and 10 unaffected individuals (Fig 1). For the proband, the wall of the carotid artery was thickened and the lumen was narrowed. His whole heart was enlarged, the thickness of each ventricular wall was decreased, and systolic function of the heart had declined visibly. The left ventricular ejection fraction was 0.40. Although the proband had taken statins and Ezetimibe, his plasma cholesterol and LDLC still did not achieve the target value.

We also recruited 100 unrelated Chinese individuals as study controls. Hypercholesterolemia-related examinations were administered by the Affiliated Hospital of Qingdao University. Peripheral blood samples were collected for DNA analysis. Informed consent was obtained from all participants. The research was consistent with the tenets of the Declaration of Helsinki and approved by the Affiliated Hospital of Qingdao University. Written informed consent was obtained from each patient, and we obtained written informed consent from the guardians on behalf of the children (Fig 1, individual IV-3) enrolled in our study.

### Mutation screening and sequence analysis

We extracted genomic DNA from 500 μL of peripheral blood using a TIANamp Blood DNA Midi Kit (Tiangen, Beijing, China). After performing genomic polymerase chain reaction (PCR), we sequenced the coding exons and their flanking intronic sequences of *LDLR* (GenBank NM_000527.4) for pathogenic mutations in the family members. The primers used in PCR have been described in an earlier report [13]. We screened for mutations in *LDLR* by direct sequencing. To verify the mutation, we separated heterozygous alleles by cloning the affected fragment into an EGFP-N1 vector. The fragment was amplified by PCR using forward primer 5′-TGAAATCTCGATGGAGTGGGTCCCATC-3′ and reverse primer 5′-CTGTAGCTAGACCAAAATCACCTATTTTTACTG-3′ and then cloned into EGFP-N1 vector. Plasmids were extracted from colonies and sequenced using the same primer. To confirm the novel mutation in *LDLR*, we also examined the mutation in the 100 unrelated controls. We performed the analysis of amino acid conservation around the mutation site using a CLC DNA Workbench (QIAGEN Bioinformatics, Germany).

### Lentivirus construction and infection

We amplified *LDLR* cDNA by PCR using the forward primer 5′-GCAGGTCGACTCTAGAGGATCGCCACCATGGGGCCCTGGGGCTGGAAATTG-3′ and reverse primer 5′-ATAGCGCTACCCGGGGATCCCGCCACGTCATCCTCCAGACTGAC-3′. The *LDLR* fragment was cloned into a BamH1 site of GV416 lentivirus vector (Genechem, Shanghai, China) using an In-Fusion Cloning Kit (Takara, Dalian, China). The mutant construct was obtained by site-directed mutagenesis. The recombinant lentivirus with the *LDLR* coding sequence was produced by co-transfection of 293T cells with the plasmids PSPAX2 and PMD2G using Lipofectamine 3000 (Invitrogen, Carlsbad, CA, USA). Lentivirus-containing supernatant was harvested 72 h after transfection and filtered through 0.22 μm cellulose acetate filters (Millipore, Billerica, MA, USA). We concentrated the recombinant lentiviruses by ultracentrifugation (2 h at 50,000 × g).

Lentivirus was transduced into the hepatoma cell line Hep G2 using the cationic polymer, Polybrene (8 mg/ml; Sigma, St. Louis, MO, USA). We obtained stable clones using antibiotic selection for two weeks. The control lentiviral transfer vector, designated Lenti-GFP, stably expressed GFP, whereas the *LDLR* lentiviral transfer vector, Lenti-GFP-LDLR, stably expressed GFP and LDLR. In our experiment, all cells were divided into three groups and designated control (transduced with GFP only), WT (transduced with GFP and wild type *LDLR*), and Mut groups (transduced with GFP and mutant *LDLR*).

### Membrane protein analysis

Briefly, we washed the cells twice with a phosphate-buffered solution (PBS) containing 1 mM magnesium chloride and 1.3 mM calcium chloride (PBS2+) and incubated for 1 h at 4°C with 0.25 mg/ml Sulfo-NHS-SS-Biotin (Pierce, Dallas, USA) diluted in PBS2+. The cells were harvested and lysed in RIPA buffer (1% NP-40, 0.5% deoxycholate, 0.1% SDS) (Beyotime, Shanghai, China) containing a protease inhibitor cocktail (Roche, Basel, Switzerland) with brief sonication. After centrifugation at 12,000 × g for 10 min, we incubated the supernatant for 1 h with streptavidin agarose (Pierce) at room temperature, followed by washing and incubation in 20% 2-mercaptoetha diluted in a sample buffer (2% SDS, 62.5 mM Tris-Cl, pH 6.8, 10% glycerol) for another 1 h. We then detected the surface LDLR and LDLR in the whole cell lysate (total LDLR) by Western blot using an anti-FLAG antibody (Sigma). Protein β-actin was detected as internal control using the anti-β-actin antibody (Sigma).

### Subcellular localization

Hep G2 cells stably expressing wild-type and mutant LDLR grown on glass coverslips were fixed with 4% paraformaldehyde (Sigma Aldrich, Shanghai, China), permeabilized with 0.1% Triton-X 100, and blocked with 5% bovine serum albumin (BSA). Mouse anti-FLAG antibody (M2 antibody, Sigma) and rabbit anti-Calnexin (CST) antibody incubations were carried out in 5% BSA at a dilution of 1:200. After incubation, we washed the cells five times with PBS, followed by incubation with Cy5 conjugated anti-mouse immunoglobulin G (IgG) and cyanine dye 3 (Cy3) conjugated anti-rabbit IgG (Jackson ImmunoResearch, West Grove, PA, USA). After six final washes with PBS, coverslips were mounted in 60% glycerol. All samples were imaged using a Leica SPE confocal microscopy (Buffalo Grove, IL, USA).

### LDL uptake analysis

We incubated Hep G2 cells with 10 μg/ml DiI-LDL in medium for 2 h. After incubation, we washed the cells three times with DPBS+ 0.3% BSA and then fixed with 4% paraformaldehyde (Sigma Aldrich). We analyzed all samples using the Leica SPE confocal microscopy and analyzed the fluorescence signal using ImageJ software (https://imagej.nih.gov/ij/).

## Results

### Clinical features

The proband was a man age 52 years old (Fig 1, individual III-1), who was diagnosed with hypercholesterolemia upon suffering a myocardial infarction 15 years ago. A fast plasma test showed him to have high levels of triglycerides (TC) (9.34 mmol/L) and LDL-C (7.88 mmol/L). He also had tendinous xanthomata and corneal arcus. He had taken statins since his myocardial infarction. His mother died at the age of 41, and she had a history of chest pain. She used to have tendinous xanthomata, a frequent clinical feature of FH. No other detailed information was available. The proband’s aunt and one of his female cousins also had tendinous xanthomata and hypercholesterolemia. One of his nephews (Fig 1, individual IV-3) had tendinous xanthomas on his elbow and hypercholesterolemia lasting one year (Table 1).

**Table 1:** Clinical characteristics findings in the family members.

Subject	Sex	Age (Years)	TG (mmol/L)	TC (mmol/L)	HDL (mmol/L)	LDL (mmol/L)	Clinical Diagnosis
II4	F	68	0.9	9.79	1.44	7.59	P
III1	M	52	2.39	9.34	1.31	7.88	P
III2	F	48	2.15	5.92	0.95	4.14	N
III3	F	49	1.93	8.38	1.28	6.38	N
III5	F	48	0.76	9.52	1.92	6.88	P
III6	M	44	2	5.53	1.38	3.47	N
IV3	M	13	1.51	10.45	1.69	8.46	P

### Mutation confirmation in *LDLR*

**Fig 2:**
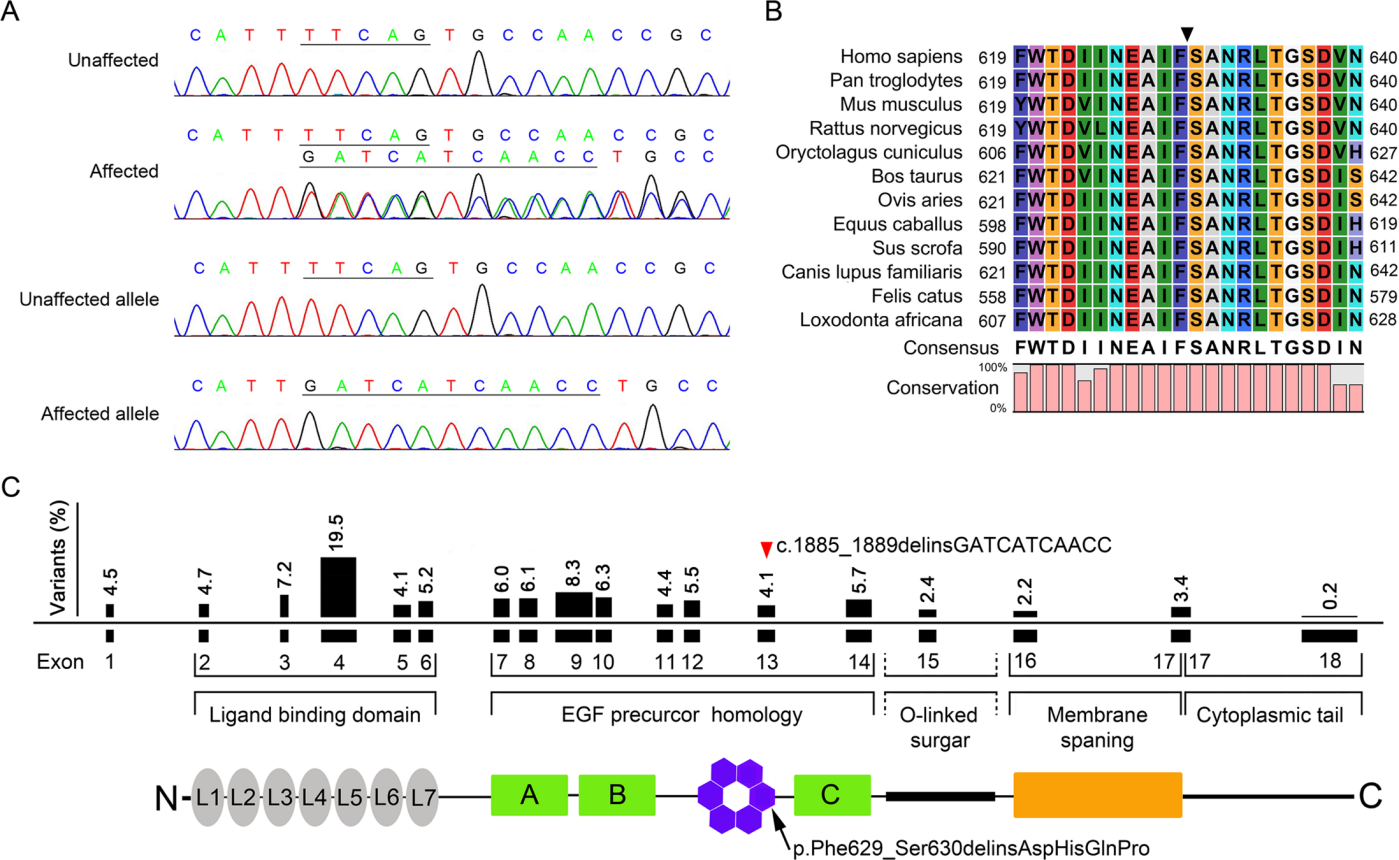
Confirmation of the c.1885_1889delinsGATCATCAACC indel mutation of *LDLR*. **(A)** Sequence chromatogram showing the c.1885_1889delinsGATCATCAACC indel mutation of *LDLR* in the proband. The mutation was numbered according to GenBank NM_000527.4. **(B)** Protein alignment of mammalian LDLR showing that the regions around the mutation are highly conserved. Numbers on left and right indicate the position of this fragment. The position of the mutation is marked by a black triangle. **(C)** Summary of the mutation responsible for familial hypercholesterolemia. The positions of the mutation in this study are marked by a red triangle and a black arrow, respectively.

A novel variant, c.1885_1889delinsGATCATCAACC, was found in exon 13 of *LDLR* by direct sequencing in the proband (Fig 2A). To confirm the mutation, we separated heterozygous alleles by cloning into the EGFP-N1 vector and sequencing (Fig 2A). The indel led to a newly formed mutant p.Phe629_ser630delinsAspHisGlnPro in the phylogenetically conserved region (Fig 2B). The only other variants detected were several nonpathogenic SNPs. The mutation was confirmed in all affected individuals but was not detected in unaffected family members or in 100 unrelated Chinese controls. There are no hot-spot mutations in *LDLR*, although the ratio of the variants in exon 4 was relatively high. The mutation in the present study was located in the sixth class B repeat, which is a class 2 mutation responsible for FH (Fig 2C). The functional characterization of the mutation in LDLR was investigated in the following study.

### The indel mutation causes defection in LDLR trafficking

**Fig 3:**
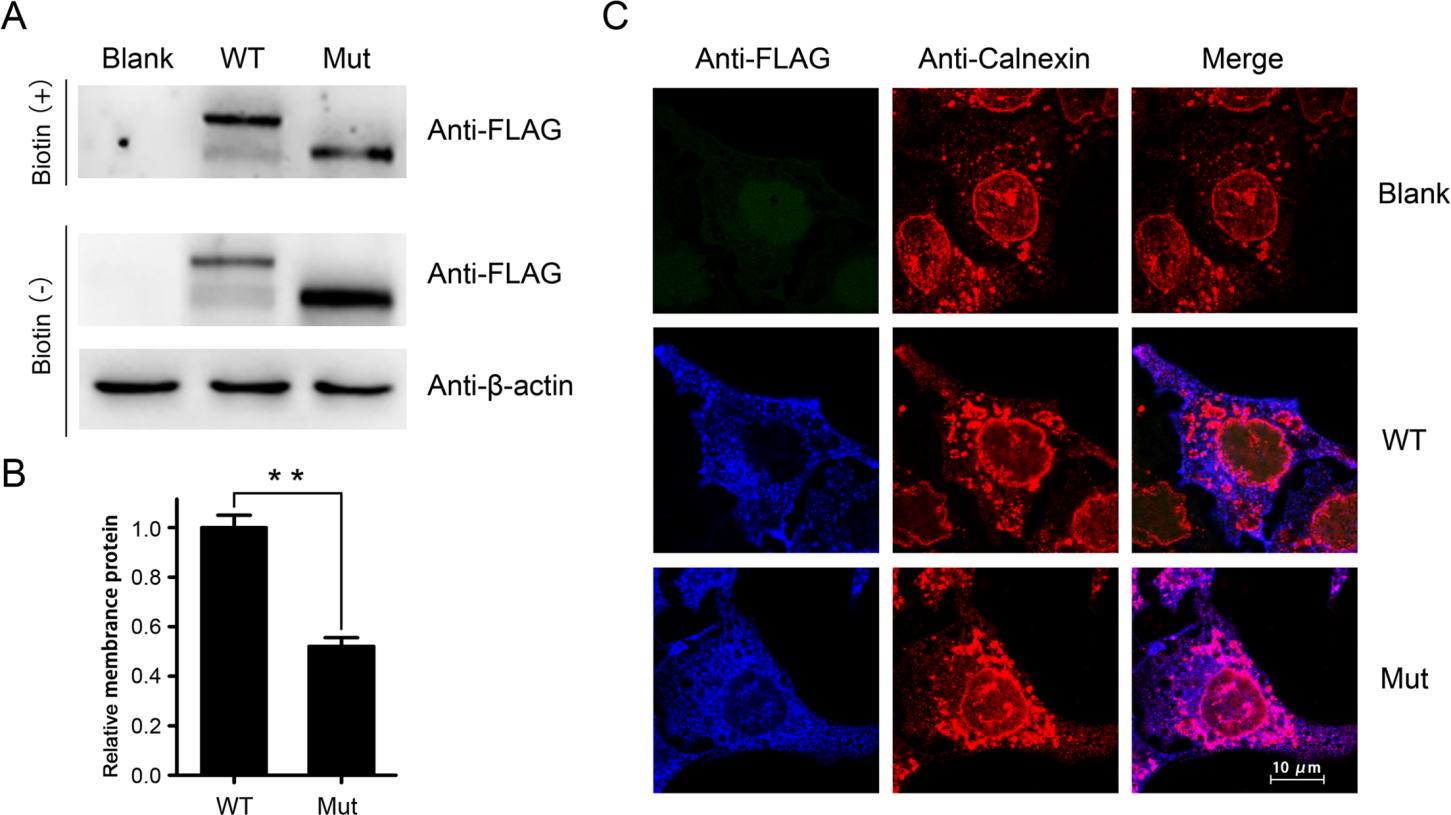
The indel disrupts the glycosylation of LDLR. **(A)** The wild-type and mutant LDLR in the membrane were labeled with biotin. Then the biotin was precipitated using streptavidin-agarose beads and immunoblotted with anti-FLAG antibodies. β-actin was used as a loading control. **(B)** Relative quantification of wild-type and mutant LDLR in the membrane were analyzed using Quantity One 1-D software (Bio-Rad, Hercules, CA, USA). Data was represented as mean value±SD (**p<0.005). **(C)** Subcellular localization of wild-type and mutant LDLR in Hep G2 cells. Calnexin is used as an endoplasmic reticulum marker.

To confirm the novel class 2 mutation in *LDLR*, we established lentivirus constructs of *LDLR*. FLAG-tagged wild type and mutant LDLR were expressed in Hep G2 cells and detected using anti-FLAG antibody. Western blotting showed that the molecular weight of recombinant mutant LDLR was 40 kD less than the wild type (Fig 3A). The mutant LDLR in the membrane was also reduced compared to the wild type (Fig 3A, Fig 3B). This suggested that the mutation may have affected the glycosylation of LDLR. We also investigated the subcellular localization of recombinant LDLR. The results showed that the mutant LDLR was stuck in ER and failed to move to the cell membrane (Fig 3C). Taken together, our findings suggest that the mutant LDLR could not be glycosylated in the Golgi apparatus and that it was retained in the ER. As a result, the molecular weight of mutant LDLR was lower than that of the wild type and less protein was transported to the cell membrane.

### Mutant LDLR depresses the uptake of LDL

**Fig 4:**
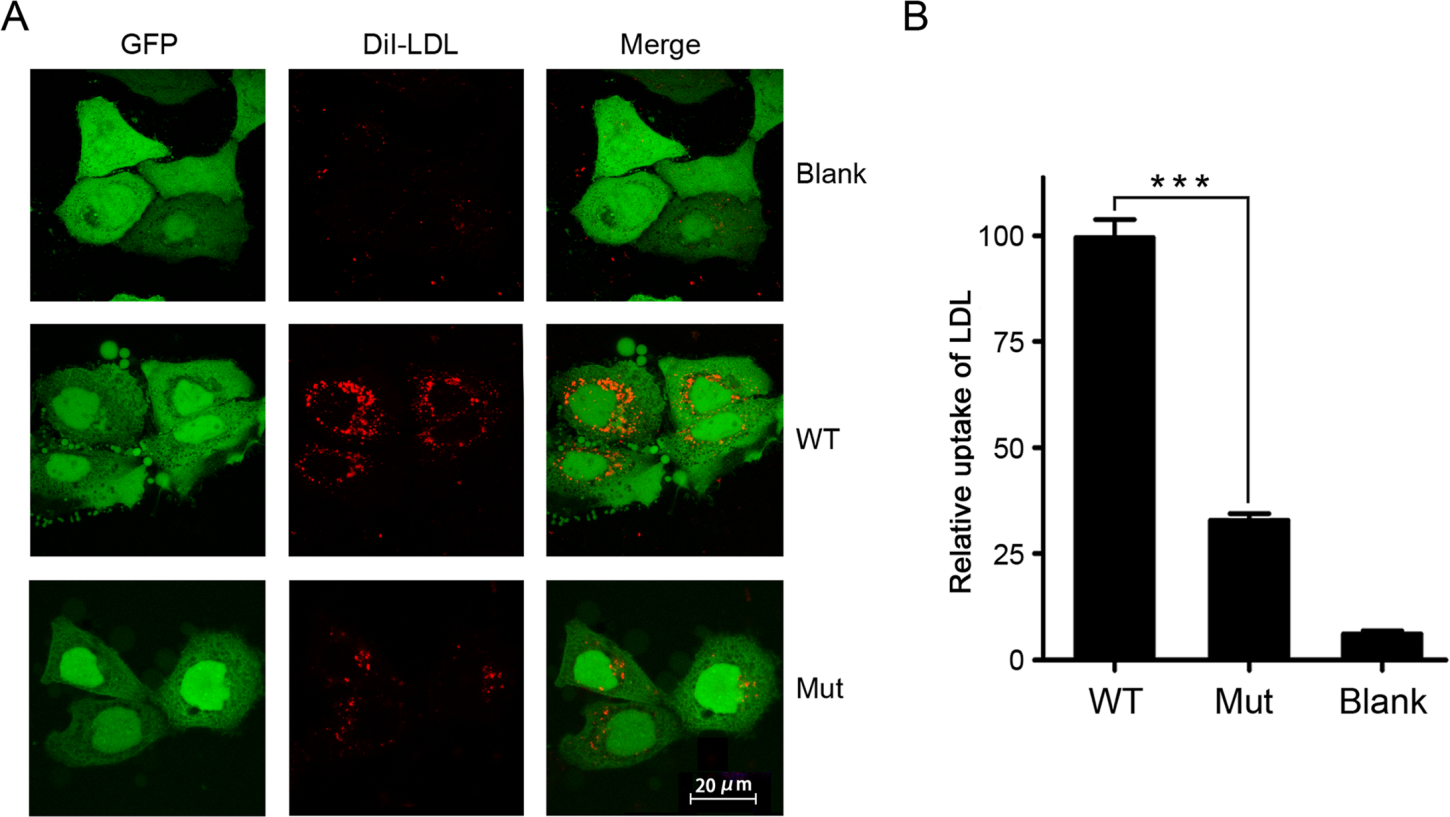
Mutant LDLR depresses the uptake of LDL. **(A)** Hep G2 cells, stably transfected with wild-type or mutant LDLR, were incubated with DiI-LDL and analyzed by fluorescence microscopy. **(B)** Fluorescence of DiI-LDL was calculated by ImageJ. One hundred cells were counted in each group and the relative fluorescence was averaged. Data was represented as mean value±SD (***p<0.001).

To analyze the pathogenicity of the mutation, p.Phe629_ser630delinsAspHisGlnPro, we performed a LDL uptake assay to assess the function of mutant LDLR. The mutation in FLAG-tagged LDLR predominantly disrupted the uptake of LDL (Fig 4A). The mutant retained 30% activity relative to the wild type (Fig 4B). Our results confirmed that the novel loss-of-function mutation in *LDLR* was the pathogenic cause of the FH in the Chinese family.

## Discussion

FH is usually considered an autosomal dominant disorder [1, 14]. It occurs in two clinical forms: homozygous and heterozygous. Homozygous FH accounts for only a small portion of cases. Among the pathogenic genes, mutations in *LDLR* are the major causes of FH [15, 16]. To date, there have been more than 1,288 different variants reported in patients with FH. Most of the mutations are exonic substitutions and no hot spots have been found [17]. About 20% of mutations are not located in the exon region. In this paper, we report a novel FH-associated indel mutation in *LDLR*, which disrupted trafficking of LDL receptor. The mutant LDLR was stuck in the ER and the protein transported to the membrane was reduced. As a result, the mutant LDLR lost the function of uptake LDL.

LDLR is a cell surface receptor mainly expressed in bronchial epithelial cells and in the adrenal gland and cortex tissue [18]. In liver cells, LDLR is inserted into the cell membrane and regulates the LDL by endocytosis of cholesterol-rich LDL [19, 20]. LDLR undergoes post-translational modifications in the Golgi apparatus whereby O-linked sugars are added. As a result, the molecular weight of LDLR increases from 120 to 160 kDa [21]. The indel mutation identified in the present study resulted in the production of an approximate 120 kDa mutant protein. We speculate that the mutant protein fails to undergo glycosylation, which may be caused by the defect in ER-to-Golgi trafficking.

*LDLR* mutations can be divided into five classes based on the biochemical and functional studies of LDLR [22]. Class 1 mutations prevent the synthesis of undetectable LDLR. Class 2 mutations cause the mutant LDLR to block completely (class 2A) or partially (class 2B) block the ER. Class 3 mutations cause the LDLR to fail to bind LDL. Class 4 mutations result in mutant LDLR that cannot internalize LDL. The LDLR with class 5 mutations fail to release LDL into the endosome [23, 24]. The mutation in the present study encoded LDLR protein with defective transport from the ER to the Golgi apparatus, which was partially blocked (Fig 3C). It decreased the amount of LDLR in the membrane and the endocytosis of LDL.

The proband showed only moderately high levels of cholesterol, for which he had taken Atorvastatin at 40 mg per day and Ezetimibe at 10 mg per day. He also followed a special diet and gave up smoking. The proband, the proband’s aunt, one of his female cousins, and one of his nephews all had tendinous xanthomata on their elbows. Except for the proband, none of them had a corneal arcus.

In Conclusion, we identified a novel indel mutation, c.1885_1889delinsGATCATCAACC, in the FH family. It was a class 2 mutation that led to the mutant LDLR blockage in ER and the endocytosis of LDL was reduced. We expanded the spectrum of *LDLR* mutations, and the indel should be considered a novel candidate mutation site causing FH.

## References

[1] Hobbs HH, Brown MS, Goldstein JL. Molecular genetics of the LDL receptor gene in familial hypercholesterolemia. Human mutation. 1992;1(6):445–66. doi: 10.1002/humu.1380010602 1301956 10.1002/humu.1380010602

[2] Pullinger CR, Hennessy LK, Chatterton JE, Liu W, Love JA, Mendel CM, et al Familial ligand-defective apolipoprotein B. Identification of a new mutation that decreases LDL receptor binding affinity. The Journal of clinical investigation. 1995;95(3):1225–34. doi: 10.1172/JCI117772 7883971 10.1172/JCI117772 PMC441461

[3] Davis CG, Lehrman MA, Russell DW, Anderson RG, Brown MS, Goldstein JL. The J.D. mutation in familial hypercholesterolemia: amino acid substitution in cytoplasmic domain impedes internalization of LDL receptors. Cell. 1986;45(1):15–24. 3955657 10.1016/0092-8674(86)90533-7

[4] Stein EA, Gipe D, Bergeron J, Gaudet D, Weiss R, Dufour R, et al Effect of a monoclonal antibody to PCSK9, REGN727/SAR236553, to reduce low-density lipoprotein cholesterol in patients with heterozygous familial hypercholesterolaemia on stable statin dose with or without ezetimibe therapy: a phase 2 randomised controlled trial. The Lancet. 2012;380(9836):29–36. 10.1016/S0140-6736(12)60771-5 22633824

[5] Awan Z, Choi HY, Stitziel N, Ruel I, Bamimore MA, Husa R, et al APOE p.Leu167del mutation in familial hypercholesterolemia. Atherosclerosis. 2013;231(2):218–22. doi: 10.1016/j.atherosclerosis.2013.09.007 24267230 10.1016/j.atherosclerosis.2013.09.007

[6] Marduel M, Ouguerram K, Serre V, Bonnefont-Rousselot D, Marques-Pinheiro A, Erik Berge K, et al Description of a large family with autosomal dominant hypercholesterolemia associated with the APOE p.Leu167del mutation. Human mutation. 2013;34(1):83–7. doi: 10.1002/humu.22215 22949395 10.1002/humu.22215 PMC3638718

[7] Garcia CK, Wilund K, Arca M, Zuliani G, Fellin R, Maioli M, et al Autosomal recessive hypercholesterolemia caused by mutations in a putative LDL receptor adaptor protein. Science. 2001;292(5520):1394–8. doi: 10.1126/science.1060458 11326085 10.1126/science.1060458

[8] Vallejo-Vaz AJ, Kondapally Seshasai SR, Cole D, Hovingh GK, Kastelein JJ, Mata P, et al Familial hypercholesterolaemia: A global call to arms. Atherosclerosis. 2015;243(1):257–9. doi: 10.1016/j.atherosclerosis.2015.09.021 26408930 10.1016/j.atherosclerosis.2015.09.021

[9] Civeira F. Guidelines for the diagnosis and management of heterozygous familial hypercholesterolemia. Atherosclerosis. 2004;173(1):55–68. doi: 10.1016/j.atherosclerosis.2003.11.010 15177124 10.1016/j.atherosclerosis.2003.11.010

[10] Heath KE, Gahan M, Whittall RA, Humphries SE. Low-density lipoprotein receptor gene (LDLR) world-wide website in familial hypercholesterolaemia: update, new features and mutation analysis. Atherosclerosis. 2001;154(1):243–6. 11137106 10.1016/s0021-9150(00)00647-x

[11] Leigh SE, Foster AH, Whittall RA, Hubbart CS, Humphries SE. Update and analysis of the University College London low density lipoprotein receptor familial hypercholesterolemia database. Annals of human genetics. 2008;72(Pt 4):485–98. doi: 10.1111/j.1469-1809.2008.00436.x 18325082 10.1111/j.1469-1809.2008.00436.x

[12] Leren TP. Sorting an LDL receptor with bound PCSK9 to intracellular degradation. Atherosclerosis. 2014;237(1):76–81. doi: 10.1016/j.atherosclerosis.2014.08.038 25222343 10.1016/j.atherosclerosis.2014.08.038

[13] Garcia-Garcia AB, Real JT, Puig O, Cebolla E, Marin-Garcia P, Martinez Ferrandis JI, et al Molecular genetics of familial hypercholesterolemia in Spain: Ten novel LDLR mutations and population analysis. Human mutation. 2001;18(5):458–9. 10.1002/humu.1218 11668640

[14] Cuchel M, Bloedon LT, Szapary PO, Kolansky DM, Wolfe ML, Sarkis A, et al Inhibition of microsomal triglyceride transfer protein in familial hypercholesterolemia. The New England journal of medicine. 2007;356(2):148–56. doi: 10.1056/NEJMoa061189 17215532 10.1056/NEJMoa061189

[15] Huijgen R, Kindt I, Defesche JC, Kastelein JJ. Cardiovascular risk in relation to functionality of sequence variants in the gene coding for the low-density lipoprotein receptor: a study among 29 365 individuals tested for 64 specific low-density lipoprotein-receptor sequence variants. European heart journal. 2012;33(18):2325–30. doi: 10.1093/eurheartj/ehs038 22390909 10.1093/eurheartj/ehs038

[16] Mollaki V, Progias P, Drogari E. Familial hypercholesterolemia in Greek children and their families: genotype-to-phenotype correlations and a reconsideration of LDLR mutation spectrum. Atherosclerosis. 2014;237(2):798–804. doi: 10.1016/j.atherosclerosis.2014.09.031 25463123 10.1016/j.atherosclerosis.2014.09.031

[17] Usifo E, Leigh SE, Whittall RA, Lench N, Taylor A, Yeats C, et al Low-density lipoprotein receptor gene familial hypercholesterolemia variant database: update and pathological assessment. Annals of human genetics. 2012;76(5):387–401. doi: 10.1111/j.1469-1809.2012.00724.x 22881376 10.1111/j.1469-1809.2012.00724.x

[18] Francke U, Brown MS, Goldstein JL. Assignment of the human gene for the low density lipoprotein receptor to chromosome 19: synteny of a receptor, a ligand, and a genetic disease. Proceedings of the National Academy of Sciences of the United States of America. 1984;81(9):2826–30. 6326146 10.1073/pnas.81.9.2826 PMC345163

[19] Goldstein JL, Brown MS. The LDL receptor. Arteriosclerosis, thrombosis, and vascular biology. 2009;29(4):431–8. doi: 10.1161/ATVBAHA.108.179564 19299327 10.1161/ATVBAHA.108.179564 PMC2740366

[20] Brown MS, Goldstein JL. A receptor-mediated pathway for cholesterol homeostasis. Science. 1986;232(4746):34–47. 3513311 10.1126/science.3513311

[21] Tolleshaug H, Goldstein JL, Schneider WJ, Brown MS. Posttranslational processing of the LDL receptor and its genetic disruption in familial hypercholesterolemia. Cell. 1982;30(3):715–24. 6291781 10.1016/0092-8674(82)90276-8

[22] Hobbs HH, Russell DW, Brown MS, Goldstein JL. The LDL receptor locus in familial hypercholesterolemia: mutational analysis of a membrane protein. Annual review of genetics. 1990;24:133–70. doi: 10.1146/annurev.ge.24.120190.001025 2088165 10.1146/annurev.ge.24.120190.001025

[23] Strom TB, Tveten K, Laerdahl JK, Leren TP. Mutation G805R in the transmembrane domain of the LDL receptor gene causes familial hypercholesterolemia by inducing ectodomain cleavage of the LDL receptor in the endoplasmic reticulum. FEBS open bio. 2014;4:321–7. doi: 10.1016/j.fob.2014.03.007 24918045 10.1016/j.fob.2014.03.007 PMC4048843

[24] Wang H, Xu S, Sun L, Pan X, Yang S, Wang L. Functional characterization of two low-density lipoprotein receptor gene mutations in two Chinese patients with familial hypercholesterolemia. PloS one. 2014;9(3):e92703 doi: 10.1371/journal.pone.0092703 24671153 10.1371/journal.pone.0092703 PMC3966815

